# Ultrafast plasma dynamics in laser-irradiated nanowire arrays probed with an X-ray free-electron laser

**DOI:** 10.1038/s41598-026-47126-0

**Published:** 2026-04-01

**Authors:** Daisuke Tanaka, Hiroshi Sawada, Chiharu Nakatsuji, Sota Matsuura, Tomoyuki Idesaka, Takumi Sato, Takuya Honda, Ichiro Nishii, Shun Horimoto, Yoshiki Takeshima, Toshihiro Somekawa, Toshinori Yabuuchi, Kohei Miyanishi, Keiichi Sueda, Yuichi Inubushi, Yasuhiko Sentoku, Tomohiro Shimizu, Shoso Shingubara, Norimasa Ozaki, Kohei Yamanoi, Keisuke Shigemori

**Affiliations:** 1 Institute of Laser Engineering, The University of Osaka, 2-6 Yamada-oka, Suita, 565-0871 Osaka Japan; 2 University of Nevada Reno, Reno, NV 89557 USA; 3 Institute for Laser Technology, 2-6 Yamada-oka, Suita, 565-0871 Osaka Japan; 4 Japan Synchrotron Radiation Research Institute, 1-1-1 Kouto, Sayo-cho, Sayo-gun, Hyogo, 679-5198 Japan; 5 RIKEN SPring-8 Center, 1-1-1 Kouto, Sayo-cho, Sayo-gun, Hyogo, 679-5148 Japan; 6 Graduate School of Science and Engineering, Kansai University, 3-3-35 Yamate-cho, Suita, 564-8680 Osaka Japan; 7 Graduate School of Engineering, The University of Osaka, 2-1 Yamada-oka, Suita, 565-0871 Osaka Japan; 8 Present Address: EX-Fusion Inc, 2-8 Yamada-oka, Suita, 565-0871 Osaka Japan

**Keywords:** Materials science, Nanoscience and technology, Physics

## Abstract

Nanowire arrays are excellent nanostructured target materials for high-energy density (HED) science and applications because of their enhanced energy absorption properties. However, investigations of the spatiotemporal dynamics of laser-irradiated nanowire arrays remain limited, since conventional time-resolved diagnostics cannot capture the rapid plasma-state transitions. This study reports spatiotemporally resolved measurements of laser energy absorption and electron transport in laser-irradiated nanowire arrays using an X-ray free-electron laser (XFEL). The XFEL measurements showed that the nanowire array is promptly heated to an electron temperature of ~ 120 eV at the main-pulse interaction peak, followed by a further increase to ~ 140 eV around 10 ps, which was associated with wire collapse. The experimental results also confirmed that further enlargement of the heated area was suppressed by the restricted electron transport in nanowire arrays. These observations advance our understanding of HED plasma formation and evolution within the laser-irradiated nanowire arrays, laying a foundation for various applications.

## Introduction

The development of ultrahigh-intensity lasers^1^, which typically deliver energy at an intensity of ~ 10^18^ Wcm^− 2^ in pulses for a few tens of femtoseconds, has made it possible to create laboratory plasmas under extreme temperature and density conditions. This drives a broad spectrum of research and applications, including inertial-confinement fusion^2,3^, laboratory astrophysics^4^, and particle acceleration^5^. In ultrahigh-intensity laser–matter interactions, a weak pre-pulse preceding the main high-intensity pulse can generate low-density pre-plasma before the arrival of the peak intensity. The subsequent peak pulse then couples not to the initial solid-density interface but mainly near the critical-density surface of this pre-plasma via mechanisms that depend on the pre-plasma scale length^6^ and laser conditions (namely, temporal contrast^7^, angle of incidence^8^, and intensity^9^. Consequently, for simple planar targets, only a smaller fraction of the laser energy is deposited into the near-solid-density material^10^. Various approaches have been proposed to enhance the laser energy absorption, including temporal pulse shaping and incidence-angle control, as well as the use of “structured targets” with sub-wavelength modulations. In such structured targets, nanometer-scale vacuum gaps and modulated interfaces can strongly modify the local laser fields, for example, by trapping and guiding the light and thereby increasing the effective optical path length and interaction time in the target. Previous studies have reported enhanced laser energy absorption using structured targets, including gratings^11,12^, nanotubes^13,14^, nanospheres^15^, and vertically aligned nanowire arrays on substrates^10,16–30^. Among these, nanowire arrays occupy a particularly favorable regime for high-energy-density applications. Their high aspect ratio provides deep vacuum gaps along which the laser field can propagate, and they have near-solid average density^10^. This combination makes it easier to create and sustain extreme states of matter.

**Fig. 1:**
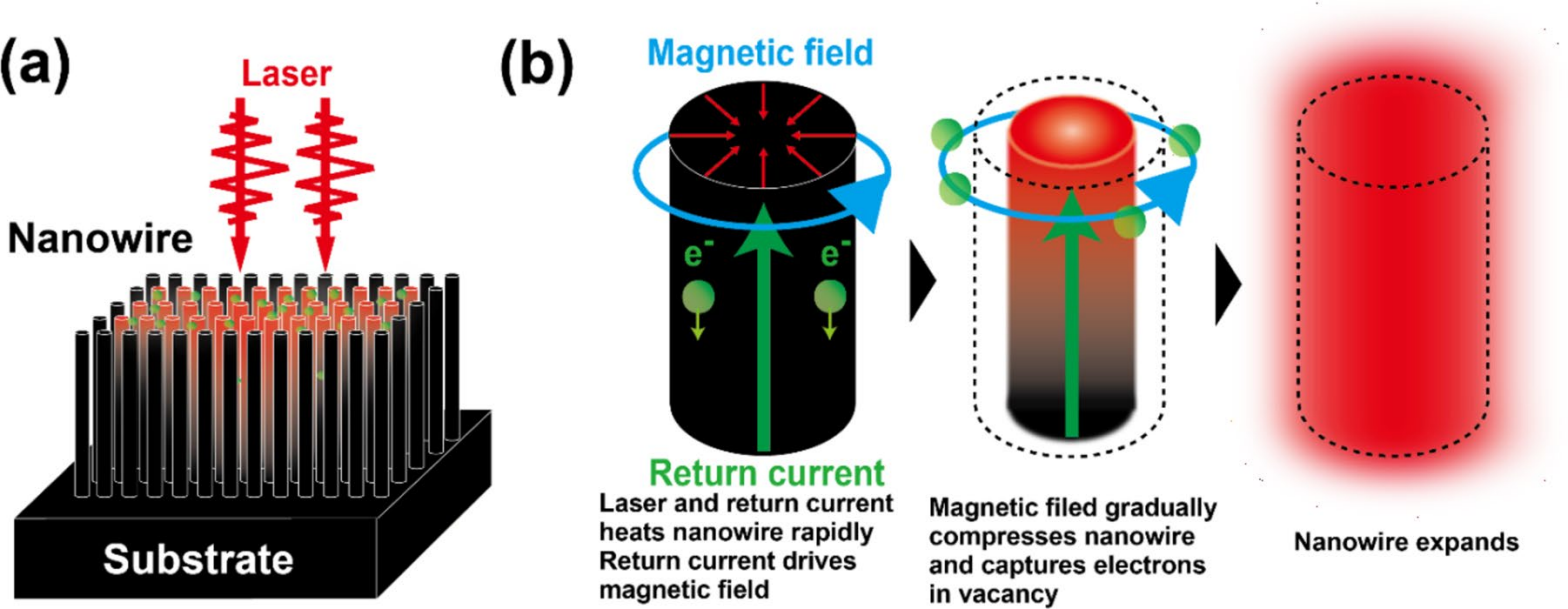
(**a**) Schematic of the interaction between an ultrahigh-intensity laser and a nanowire array. It is worth noting that this schematic viewgraph does not represent the actual experimental setup used in this study. (**b**) Temporal evolution of the laser-irradiated nanowire. In the initial phase, the nanowires are heated by the laser and return current promptly. This return current drives the intense magnetic field, which pinches the nanowire and captures the electrons in the vacancy between nanowires. Finally, the nanowire array plasma expands. Black dot pillar in (b) represents the initial nanowire shape.

Figure 1a shows the schematic of a laser-irradiated nanowire array. The laser can penetrate deeper into the gaps between the high-aspect-ratio nanowires than into a flat foil, allowing more efficient energy absorption in the nanowire array^10^. Figure 1b illustrates the dynamics and temporal evolution of the thermal energy in the laser-irradiated nanowire array. Hot electrons in the nanowire cannot move freely to adjacent nanowires because of the inter-nanowire gap. Instead, these electrons move toward the substrate and drive a strong return current^18^ that induces a strong quasi-static magnetic field around each nanowire^18,19^. This confines the laser-driven hot electrons and suppresses the lateral energy spread^20^. Subsequently, the nanowires explode, thereby increasing the electron temperature of the system^21^. The resulting hot plasma is sustained for an extended period because of its longer hydrodynamic cooling time compared to its radiative cooling time (~ an order of magnitude)^22^. In the final phase, the plasma expands. These phenomena enable efficient energy absorption within the laser-irradiated nanowire arrays and are expected to provide access to fascinating high-energy density applications. For example, Eftekhari-Zadeh et al. ^23^ reported an average laser energy absorption of ~ 80%, reaching up to 86%, in ZnO nanowire arrays, compared with ~ 40% in flat foils. Utilizing the extremely compressed nanowires with high electron temperatures, deuterium–deuterium fusion reactions can be achieved with nanowires made of deuterated polyethene^24,25^. Hollinger et al. ^22^ achieved a record optical-to-x-ray conversion efficiency of ≈ 18% on average, with single shots reaching up to 22% into *hν* > 1 keV photons, using laser-irradiated Au nanowire arrays. Understanding the underlying heating and transport mechanisms in nanowire arrays is crucial for optimizing ultrahigh-energy applications^22,24–27^.

Although various studies have investigated energy absorption and transport in laser-irradiated nanowire arrays^28–30^, most previous studies have inferred the plasma parameters within laser-irradiated nanowire arrays indirectly by comparing time-integrated X-ray spectra and accelerated-particle spectra with particle-in-cell (PIC) simulations. Such diagnostics and modelling provide valuable constraints; however, they only infer the dynamics inside the nanowire arrays rather than directly resolving them. It is difficult for conventional time-resolved instruments to observe fast and small-scale phenomena originating from ultrahigh-intensity laser irradiation with a short laser pulse (a few tens of femtoseconds) and a small focal spot (a few tens of micrometers). In a pioneering work, Humphries et al.^21^ employed an ultrafast X-ray streaked spectrometer to observe X-ray self-emission from nanowire arrays irradiated by a picosecond, kilojoule-class laser, revealing transient plasma behavior that agreed with simulations. However, the temporal resolution of approximately 1 ps and the poor spatial information from these experiments are insufficient for observing the ultrafast temporal evolution of thermal energy in laser-irradiated nanowire arrays. In particular, the spatiotemporal evolution of electron temperature and geometry has not been directly measured inside laser-irradiated nanowire arrays with simultaneous sub-picosecond temporal and micrometer-scale spatial resolution.

X-ray free-electron lasers (XFELs) have emerged as an ultrafast probe source. They deliver ultrashort, high-brightness X-ray pulses and are employed in plasma physics^31,32^, condensed-matter physics^33,34^, processing^35^, and materials science^36,37^. Recently, Sawada et al.^31,32^ conducted time-resolved measurements on the laser-irradiated thin Cu foil at the SPring-8 Angstrom Compact free electron Laser (SACLA) facility^38,39^. Building on this capability, we herein report the comprehensive plasma dynamics inside a laser-irradiated nanowire array observed by ultrafast pump–probe measurements using XFEL.

In this study, we used an ultrahigh-intensity optical laser as a pump laser and an XFEL pulse as a probe to perform an ultrafast shadowgraph of laser-irradiated nanowire arrays. We observed the temporal evolution of the plasma within the laser-irradiated nanowire arrays by changing both the pump–probe delay and the XFEL photon energy. In the early phase, the nanowire array was rapidly heated; after 10 ps, the electron temperature increased because of the collapse of the nanowires. The heated area did not expand because the energy dissipation was suppressed. We also observed energy transport into the substrate layer and determined the hot electron transport time from the nanowire arrays to the substrate layer. By employing a two-material nanowire array whose nanowires and substrate are made of different elements, we can separate heating in the nanowires from the energy deposited in the substrate. The results indicate that it is critical to use two-material nanowire array targets for XFEL pump–probe measurements. Overall, by combining an XFEL probe with two-material nanowire targets, our measurements directly access internal plasma dynamics that had previously only been inferred. This refines the picture of laser-driven heating and helps guide future high-energy-density applications.

## Results

### Experimental setup

**Fig. 2:**
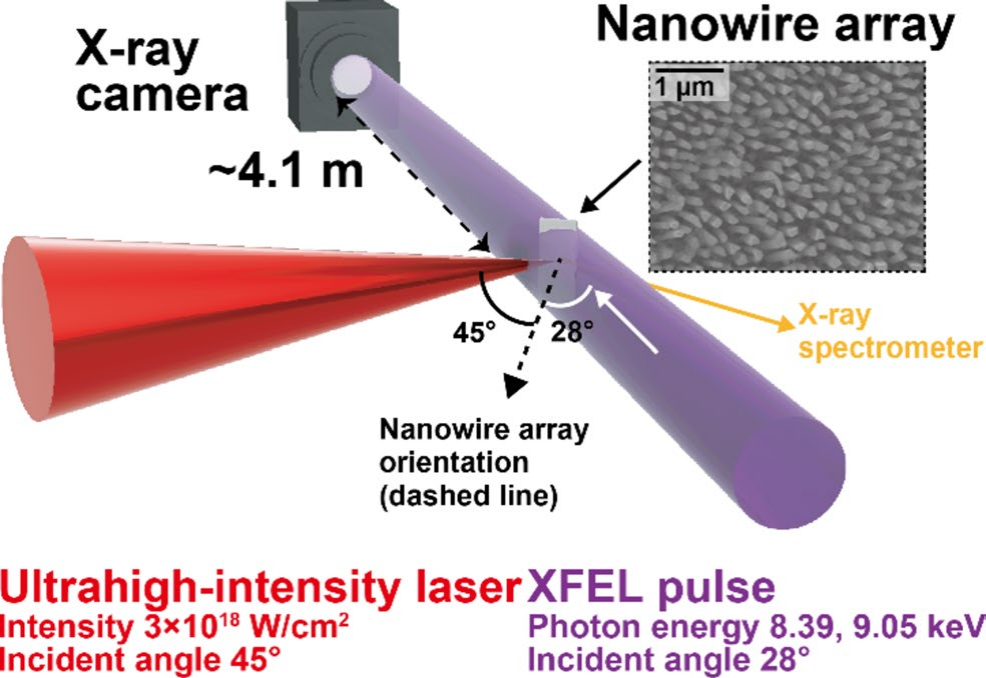
Schematic of the experimental setup for the pump-probe experiment. A tightly focused ultrahigh-intensity laser irradiated the target; then, an XFEL pulse transmitted the target with the time delay. The transmitted XFEL pulse was recorded by a CMOS camera. The self-emitted x-ray spectrum reflected from the highly annealed pyrolytic graphite crystal was recorded with a CCD camera.

Experiments were conducted at Experimental Hutch 6 (EH6) of SACLA Beamline 2 (BL2), a high-energy-density physics platform^39^. Fig. 2 shows a schematic of the experimental setup. The target was irradiated with an ultrahigh-intensity laser at a peak intensity of 3 × 10^18^ Wcm^[-2^. The pulse temporal contrast was 10^7^ and 10^10^ at 10 and 50 ps before main pulse arrival, respectively. Synchronized with an ultrahigh-intensity laser pulse, the target was probed with an XFEL pulse. The XFEL photon energy was tuned to either 8.39 or 9.05 keV. The nanowire target was probed by varying the pump–probe delay (*Δt*) between the ultrahigh-intensity laser and XFEL. Timing error was ± 0.1 ps based on the uncertainty of the XFEL and the optical laser timing jitter. XFEL shadowgraph images were recorded using a 16-bit scintillator-based CMOS camera^40^ with 20× magnification. The spatial resolution of the diagnostic system was approximately 3 µm^32^. We have also measured self-emitted x-ray from a laser-irradiated nanowire array to monitor the plasma conditions within the laser-irradiated nanowire array. X-ray spectra were observed via a Bragg crystal spectrometer using highly annealed pyrolytic graphite. Further details of the experimental configuration are provided in the Methods section.

The XFEL photon energy selected was just above the K-absorption edges of Cu (8.998 keV) and Ni (8.333 keV) to observe X-ray transmittance changes due to the increase in electron temperature *T*_e_ and ionization states *Z*. When *T*_e_ was below the Fermi temperature $\:{T}_{\mathrm{F}}=\left({\hslash\:}^{2}/2{m}_{e}{k}_{B}\right){\left(3{\pi\:}^{2}{n}_{e}\right)}^{2/3}$(Fermi degenerate state), we applied the K-edge slope diagnostics method^31,32,41,43^. For Ni which was used as the nanowire material, *T*_*F*_ ≈ 12 eV. The X-ray absorption spectrum near the K-absorption edge followed a Fermi-Dirac distribution, written as $\:{f}_{\mathrm{D}}\left(E\right)=1/\left(1+{e}^{\left(E-\mu\:\right)/k{T}_{e}}\right)$, where *µ* is the chemical potential of the target material. By setting the XFEL photon energy to a few tens of eV above the K-edge, heated areas can be observed as transmittance-affected areas. This analysis method is valid for *T*_e_ <*T*_F_^42^. When *T*_e_ > *T*_F_ (thermally and highly ionized, non-degenerate), *T*_e_ was estimated using the collisional-radiative code FLYCHK^44^. In the Ni nanowire observation, the plasma is expected to be in a high-temperature, non-degenerate state; therefore, we used FLYCHK to evaluate the *T*_*e*_ dependence of the x-ray transmittance. At 8.39 keV, just above the Ni K-absorption edge, the calculated transmittance exhibits a convex dependence on *T*_*e*_; therefore, both decreases and increases in transmittance are possible in the relevant temperature range, as discussed in the Results section. By contrast, the Cu substrate remains relatively cool and Fermi-degenerate. For the 9.05-keV probe, just above the Cu K-absorption edge, the K-edge-slope method predicts a monotonic increase in transmittance with *T*_*e*_ in the range accessed in this experiment.

We fabricated two types of nanowire array targets: Ni-on-Cu and Cu-only nanowire arrays. The Ni-on-Cu nanowire array was grown on a 5 μm-thick Cu substrate and consisted of 200 nm-diameter Ni nanowires that were 3 μm in length, with a filling factor of ~ 13%. The Cu-only nanowire array was composed of Cu nanowires of the same diameter, length, and filling factor, grown on a 5 μm-thick Cu substrate. The Ni-on-Cu nanowire array was employed to observe the X-ray transmittance change in the nanowire array and substrate layer independently. According to the CXRO database^45^, the X-ray transmittance of 5 μm-thick Cu at 8.39 keV is 0.82, minimizing attenuation by the bulk layer during Ni nanowire array observation. Both targets were fabricated using the anodic aluminum oxide template method^45^; detailed fabrication procedures are provided in the Methods section.

### Electron‑temperature evaluation and spatial mapping of the heated area in the nanowire array

**Fig. 3:**
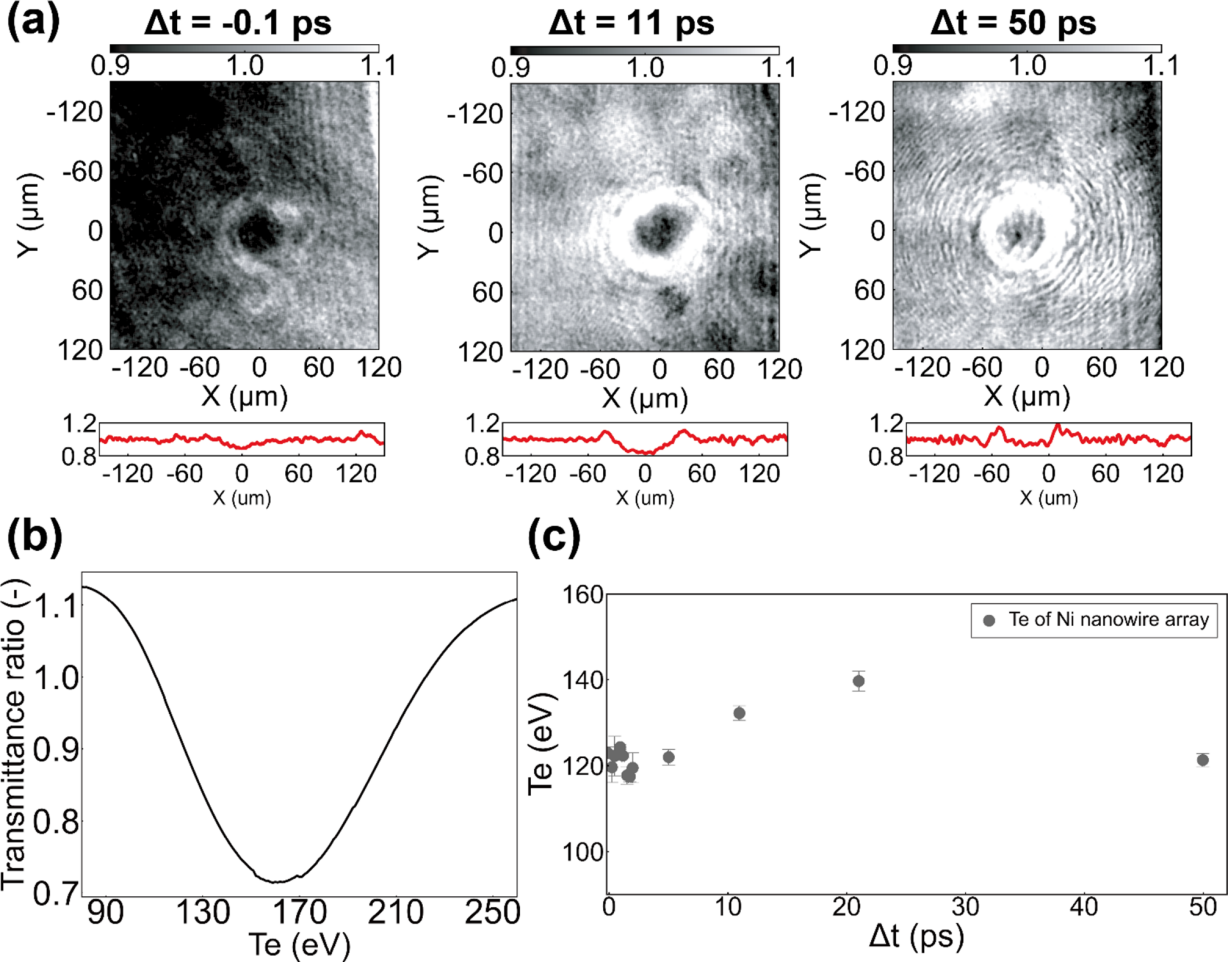
(**a**) Time-resolved XFEL transmittance–affected images at 8.39 keV XFEL irradiation for the Ni-on-Cu nanowire array at delays *Δt* = − 0.1, 11, and 50 ps, together with their corresponding line profiles. (**b**) Calculated dependence of the Ni transmittance-change ratio for a 3.4-µm-long wire segment at 13% of the solid density as a function of electron temperature calculated by FLYCHK. **(c**) Temporal evolution of the electron temperature of *Δt* = − 0.1 to 50 ps. Horizontal (time) error bars are ± 0.1 ps, set by the timing uncertainty of the XFEL and the ultrahigh intensity laser timing jitter. Vertical error bars in (c) mean shot-to-shot fluctuations in the ratio extracted from divided images.

Figure 3a shows the time series of the X-ray transmittance ratio images at 8.39 keV for the Ni-on-Cu nanowire array. The earliest image, labeled − 0.1 ps (please note that *Δt* = -0.1 ps was within the ± 0.1 ps timing uncertainty of *Δt* = 0), captures the main-pulse interaction peak. Immediately after the main-pulse interaction peak, a dark circular area was observed, indicating a decrease in X-ray transmittance. Approximately 10 ps later, the dark circular area became more pronounced, and a surrounding white ring—an area of increased transmittance—appeared. At *Δt* = 50 ps, the X-ray transmittance ratio increased and weak fringe patterns became prominent. The decrease in X-ray transmittance can be attributed to the resonant 1s → 3p (Kβ) transition. The cold Ni-Kβ line appears at 8.26 keV, but ionization increases the bound-state energies and shifts the resonant Kβ absorption to higher photon energies^43^. Figure 3b shows the transmittance ratio of Ni, calculated by FLYCHK as a function of electron temperature, for an effective path length of 3.4 μm (including the 28° XFEL incidence angle) and an average density of 13% of the solid density. When the electron temperature was between 110 and 220 eV, the X-ray transmittance decreased, indicating the creation of a highly heated area. The relatively low-temperature region formed by heat transfer from the hot central plasma is indicated by a white ring. We estimated the maximum electron temperature of the nanowire array using the minimum transmittance ratio at each *Δt* and compared it with the FLYCHK calculation results. Figure 3c shows the *T*_e_ versus *Δt* plot. Details of the minimum transmittance and *T*_e_ evaluation and error computation are provided in the Methods section. During the early phase (*Δt* = − 0.1 ps to 5 ps), the minimum ratio remained at ~ 0.9. Compared to the FLYCHK calculations, the electron temperature and ionization state were estimated to be ~ 120 eV and ~ 13, respectively, in this interval. Thus, the ultrahigh-intensity laser heated the nanowires quasi-instantaneously, and the electron temperature was maintained until *Δt* ≈ 5 ps. Later, the minimum transmittance ratio began to decrease at around *Δt* ≈ 11 ps and reached 0.78 at *Δt* ≈ 21 ps, corresponding to an electron temperature of ~ 140 eV and ionization state of ~ 14 according to FLYCHK calculations. This is due to the collapse of the nanowire array. When the nanowires began to expand, their kinetic energy was converted into thermal energy^21^. We evaluated the timescale of nanowire expansion using the speed of sound (*v*_*s*_) in Ni. For *T*_e_ ≈120 eV, Z ≈ 13 (FLYCHK calculation results), and $\:{v}_{s}=\sqrt{Z{k}_{b}{T}_{e}/{m}_{i}}$, where *k*_*b*_ is Boltzmann constant and *m*_*i*_ is ion mass, the *v*_*s*_ value of Ni was approximately 50 nm/ps. The converging and expanding waves propagate in the 200 nm diameter nanowire for 4 ps (i.e., less than 5 ps). It is likely that magnetic pinching reduces the expansion velocity of the nanowire array, because of which the onset of expansion occurs later than *Δt* = 5 ps. Our earlier work on nanowires of comparable geometry at the same laser intensity focused on the shape change of the nanowire array and also revealed the collapse of the nanowire array ∼10 ps after laser irradiation^45^. The increase in the transmittance ratio approximately 50 ps after laser irradiation indicated a decrease in the electron temperature. The fringe pattern was attributed to X-ray diffraction owing to surface deformation^31^. We also considered radiative (*τ*_*rad*_) and hydrodynamic (*τ*_*hyd*_) cooling. Using the evaluated *T*_*e*_ along with the radiative power obtained from FLYCHK, we estimated *τ*_*rad*_ to be approximately 9 ps. By contrast, *τ*_*hyd*_ was approximately 50 ps, which was obtained by dividing the plasma size (3 μm) by the speed of sound (~ 60 nm/ps, evaluated for *T*_*e*_ ≈ 140 eV and *Z* ≈ 14). This indicates that, under the present conditions, the plasma is in a radiation-loss-dominated cooling regime^22^. In addition, under the present conditions (*a*_*0*_ ≈ 1; nanowire diameter > skin depth), along with the fact that the collapse occurs on picosecond time scales, nanowire expansion is unlikely to be driven by a prompt Coulomb explosion. In addition to experimental data, we conducted PIC simulations for the interpretation of *T*_*e*_ increase associated with nanowires collapse, details are provided in the Methods. Please note that this PIC simulation was performed for qualitative comparison with the experimental results. The electron temperature obtained in the PIC simulation did not quantitatively agree with the experimentally inferred value. This discrepancy arises from multiple factors associated with the simplified simulation setup, including the absence of pre-plasma, the idealized nanowire geometry, and the use of cell-averaged electron temperature.

**Fig. 4:**
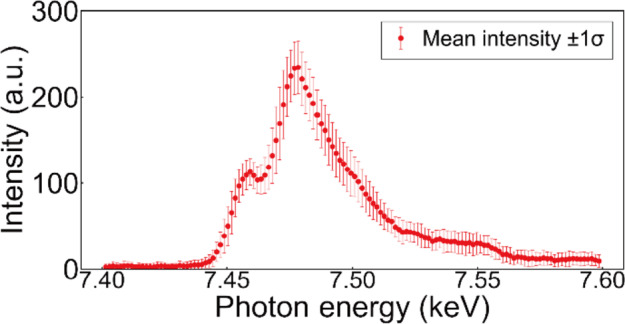
Background-subtracted, shot-averaged self-emission x-ray spectrum from the laser-irradiated Ni-on-Cu nanowire array in the Ni K-alpha region. The red error bars indicate the shot-to-shot standard deviation (± 1σ) of the measured intensity. Figure 7 shows representative raw data and the corresponding self-emitted x-ray spectrum.

We also evaluated *T*_*e*_ and *Z* via x-ray spectroscopy. Figure 4 shows a shot-averaged self-emitted x-ray spectrum along with the shot-to-shot variability (± 1σ). The spectrum exhibits a shifted Ni Kα line around 7.50 keV. As ionization progressed, the inner-shell electron configuration changed, altering the electron potential energy relative to that under ambient conditions. Consequently, the Kα line (2p to 1s) shifted toward a higher photon energy, resulting in shifted Kα emission. According to the multi-configuration Dirac–Fock calculations by Szymańska et al.^47^, the observed Kα shift to 7.50 keV (a shift of ~ 20 eV) indicates that the Ni nanowires reach a mean charge state of *Z* ~ 13–14. These ionization values are in good agreement with evaluated *T*_*e*_ and *Z* value via x-ray shadowgraph measurements. A weak feature was also observed around 7.55 keV, suggesting that a fraction of Ni atoms reached a higher ionization state with L-shell depletion. However, we did not observe strong high-energy satellite lines that would be characteristic of multi-keV plasmas seen in many previous reports^10,17,23^. This meant that the plasma was hot enough to ionize up to the L-shell, but not in the multi-keV regime reported in other works that used ultrahigh-contrast relativistic lasers.

**Fig. 5:**
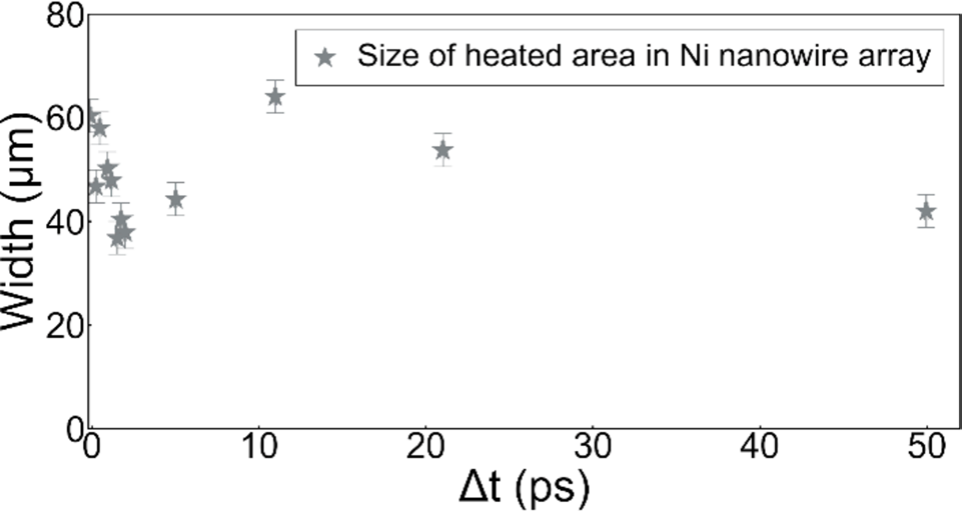
Temporal evolution of the transmittance affected area width. Each width was determined using 5% of the peak signal as the detection threshold. For details of the analysis procedure, please refer to the Data analysis section. Horizontal (time) error bars are ± 0.1 ps based on the timing uncertainty of the XFEL and the laser timing jitter. Vertical error bars are ± 3 μm, originating from the spatial resolution of the diagnostic system.

Figure 5 shows the temporal evolution of the width of the area that exhibited decreased transmittance. While expansion of the transmittance-affected area owing to hot electron transport was observed in the Cu thin foil in previous experiments^31,32^, no significant expansion was observed in the Ni nanowire. This is because the gaps between the nanowires and the intense magnetic fields surrounding each nanowire prevented hot electron propagation and energy dissipation^20^. Therefore, the plasma generated inside the nanowire array was distributed within a limited area.

### Energy transfer from the nanowire array to the underlying substrate

**Fig. 6:**
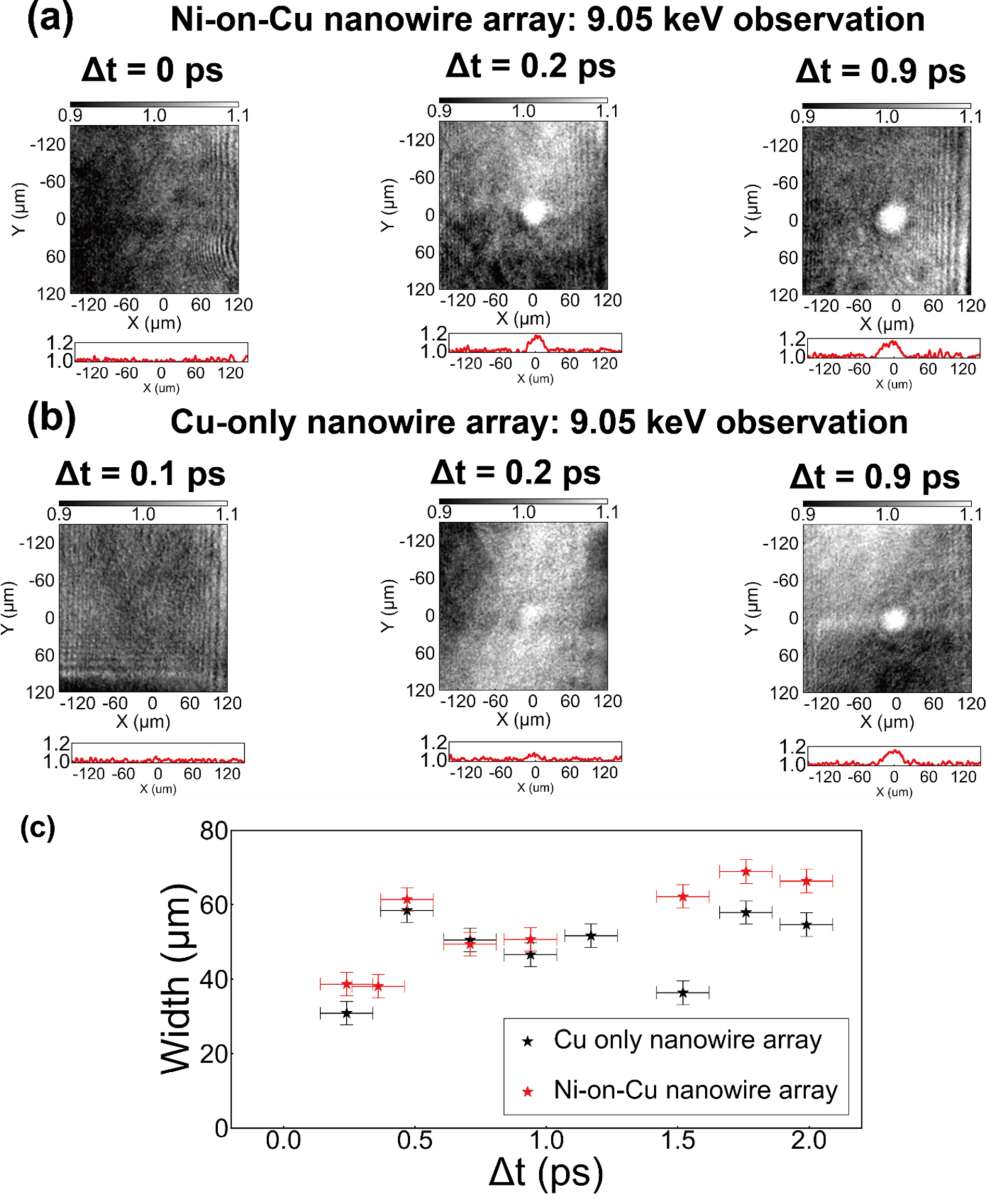
X-ray transmittance-affected images at 9.05 keV XFEL irradiation for the (**a**) Ni-on-Cu nanowire array and (**b**) Cu-only nanowire array and their corresponding line profiles at *Δt* = 0, 0.2, and 0.9 ps. (**c**) Comparison of the width of the transmittance-affected area of the Cu-only nanowire array and Ni-on-Cu nanowire array at 9.05 keV XFEL irradiation. As in Fig. 5, each width was determined using 5% of the peak signal as the detection threshold. Error bars in time are ± 0.1 ps, and those in width are ± 3 μm (as calculated from Fig. 5).

Figure 6a shows the time series of the X-ray transmittance ratio images from the 9.05 keV XFEL irradiation. At the onset of the peak of the main-pulse interaction, no change in X-ray transmittance was observed in the Cu substrate, whereas the X-ray transmittance of the Ni nanowire layer changed immediately after the onset of the main-pulse peak. After 0.2 ps of laser irradiation, the X-ray transmittance increased and the transmittance-affected area started to expand.

Based on the K-edge-slope diagnostic established by Sawada et al.^31,32^, this increase corresponded to an electron temperature of ≈ 8 eV, indicating the substrate was in the Fermi degenerate state^31,32^. The 0.2 ps time lag was the time required for the hot electrons from the Ni nanowire array to reach the underlying Cu substrate.

Figure 6b shows the time series of the X-ray transmittance ratio images at 9.05 keV XFEL irradiation for the Cu-only nanowire array. In the Cu-only nanowire array, we observed a clear increase in X-ray transmittance, consistent with the Cu substrate layer behavior in the Ni-on-Cu target. In addition, there was a 0.2 ps time lag before the X-ray transmittance increased and the expansion of the transmittance-affected area became evident. This trend was quite similar to that seen in the Ni-on-Cu nanowire array irradiated at 9.05 keV. Figure 6c plots the width of the transmittance-affected area versus *Δt* at 9.05 keV XFEL irradiation for the two cases: the Cu-only nanowire array (black points) and the Ni-on-Cu nanowire array (red points). Compared with the observations at 8.39 keV XFEL irradiation, the heated area in the Cu-only nanowire array and the Cu substrate layer of the Ni-on-Cu nanowire array expanded during the early phase and then remained constant until *Δt* ≈ 1 ps. The widths and temporal offsets observed in the Cu-only array agreed well with those of the Cu substrate in the Ni-on-Cu target. This indicated that the low-density (~ 13%) nanowire region had no measurable transmittance change in the Cu-only nanowire array because the high-density (~ 100%) Cu substrate dominated the signal. These observations indicate that it is essential to employ multimaterial targets for pump–probe measurements of laser-irradiated nanowire arrays.

## Discussion

We comprehensively examined the energy absorption and electron transport process in a laser-irradiated nanowire array by ultrafast pump–probe measurements using an ultrahigh-intensity laser and XFEL simultaneously. We observed the X-ray transmittance changes due to the increase in the electron temperature and ionization state in the nanowire and substrate layers separately by optimizing the XFEL photon energy and target configuration. The electron temperature of the Ni nanowires was estimated to be approximately 120 eV at the main-pulse interaction peak. Then, the electron temperature increased to ~ 140 eV owing to the expansion of the nanowires at around *Δt* = 10 ps. This time scale was in good agreement with the velocity of sound in Ni and with previous experimental results.

We also observed the confined plasma created in the nanowire arrays. The substrate layer was in a Fermi degenerate state, and the 0.2 ps time delay was attributed to the transport time of the hot electrons. The Cu-only nanowire array exhibited a behavior similar to the Ni-on-Cu nanowire array irradiated at 9.05 keV. This indicated that the X-ray transmittance changes in the low-density nanowire area were obscured by the density difference in the Cu-only nanowire arrays. These findings demonstrate the importance of employing a two-material nanowire array in XFEL pump–probe shadowgraph measurements.

These results provide key insights into the laser energy absorption and transport inside the laser-irradiated nanowire array and offer guidelines for developing numerous applications of such arrays, such as bright X-ray generation, particle acceleration, and microscale fusion.

## Methods

### Diagnostics

Laser irradiation experiments were conducted in Experimental Hutch 6 (EH6) at the SACLA XFEL facility. The EH6 had a high-power femtosecond Ti: sapphire laser system synchronized with an XFEL beam^39^. The wavelength of the ultrahigh-intensity laser was 800 nm, and the pulse width was 30 fs at full width at half maximum (FWHM). The focal spot size was approximately 20 μm at FWHM, and the resulting peak intensity was ≈ 3 × 10^18^ Wcm^− 2^. The laser energy used in this study was adjusted to be 1.1 ± 0.1 J; and the pulse temporal contrast was 10^7^ (i.e. ≈3 × 10^11^ Wcm^− 2^) and 10^10^ (i.e. ≈3 × 10^8^ Wcm^− 2^) at 10 and 50 ps, respectively, before the main pulse arrival. We irradiated the target with an ultrahigh-intensity laser at an angle of incidence of 45° after reflection off an f/10 off-axis parabolic mirror. Laser light was p-polarized. The ultrahigh-intensity laser specifications are summarized in Table 1. The XFEL pulse width was 10 fs at FWHM and the spot size was ~ 1 mm. The XFEL photon energy was 8.39 or 9.05 keV with a spectral bandwidth of 30–40 eV. We recorded shadowgraph images with a CMOS camera whose pixel size was 6.5 μm and which had a total of 2048 × 2048 pixels (Hamamatsu Photonics ORCA-Flash4.0 V2)^43^.

**Table 1:** Ultrahigh-intensity laser specifications.

Wavelength	800 nm
Pulse duration (FWHM)	30 fs
Energy on target	1.1 ± 0.1 J
Focal spot (FWHM)	~ 20 μm
Peak intensity	3 × 10^18^ Wcm^− 2^
Temporal contrast 50 ps before main pulse arrival	10^− 10^
Temporal contrast 10 ps before main pulse arrival	10^− 7^
Polarization	p-polarized
Incidence angle	45°

**Fig. 7:**
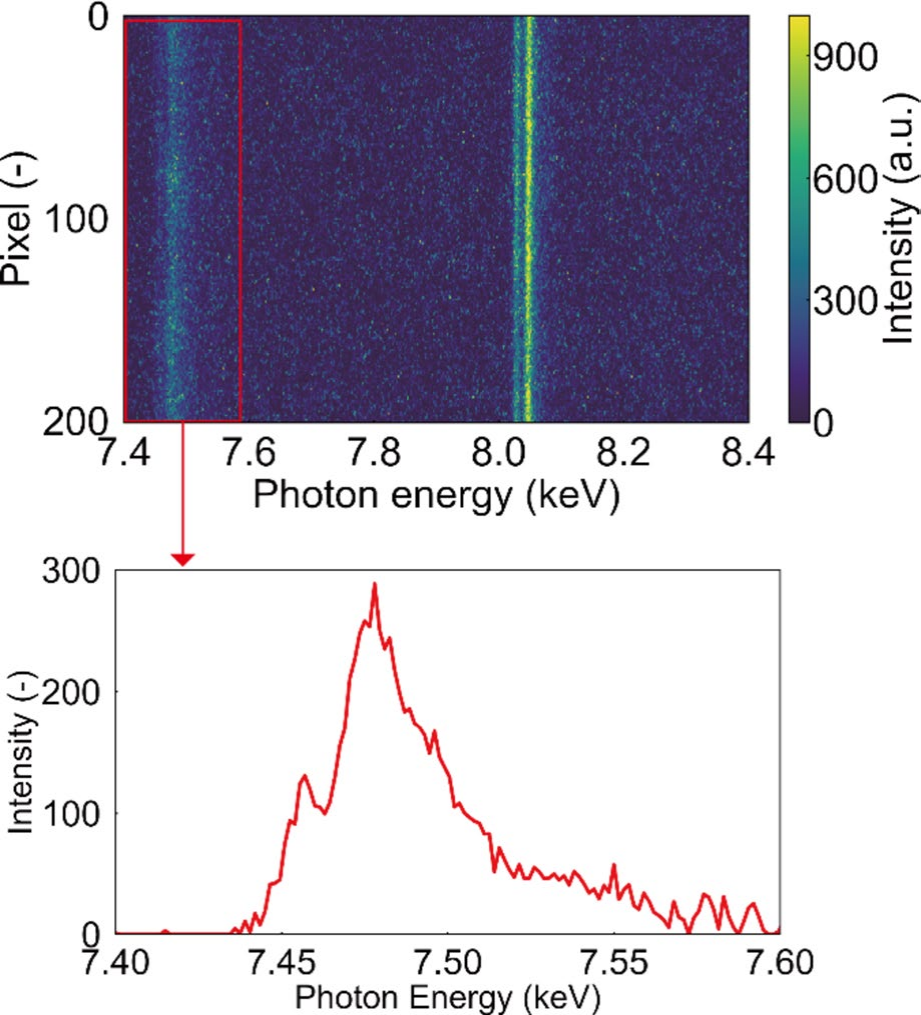
Representative single-shot self-emitted x-ray spectrum from a laser-irradiated Ni-on-Cu nanowire array obtained by x-ray crystal spectrometer measurement. Upper panel: raw two-dimensional spectrometer image; Lower panel: corresponding one-dimensional line profile obtained by averaging the signal within the red rectangle. In the line profile, the background signal was subtracted.

In the experiments, we first captured a few XFEL shadowgraphs without ultrahigh-intensity laser irradiation as background images. After capturing the background images, we irradiated the target with ultrahigh-intensity laser and obtained the shadowgraph image with the timing delay (*Δt*) between the ultrahigh-intensity laser and XFEL. We obtained the shadowgraph images with varying *Δt*, from − 0.2 to 50 ps. The shot rate in the experimental series was approximately 3 min per shot. It is worth noting that the pump–probe *Δt* was defined for each experimental run by using a Cu flat foil and identifying the delay at which the XFEL transmittance started to change. This delay was taken as *Δt* = 0, corresponding to the main-pulse interaction peak. Please note that we obtained only one x-ray transmittance ratio dataset for each *Δt*. To monitor the reproducibility of the plasma conditions in the laser-irradiated nanowire array, we simultaneously recorded the self-emission x-ray spectra using a Bragg crystal spectrometer. Self-emitted x-rays from laser-irradiated nanowire arrays were also observed through reflection from a highly annealed pyrolytic graphite crystal^39^ coupled to a CCD camera. The energy dispersion of the x-ray spectrometer was 2 eV/pixel. A representative raw x-ray spectrum and corresponding line profile are shown in Fig. 7.

### Data analysis

As described above, prior to ultrahigh-intensity laser irradiation, we irradiated the target with the XFEL only and captured several background images for each delay. After these background shots, the ultrahigh-intensity laser irradiated the target, and a signal image was acquired. To emphasize the transmittance change due to the increase in electron temperature, each signal image was divided by the corresponding background images to obtain normalized transmittance (ratio) images^31,32^, effectively flat-fielding the XFEL spatial profile. For each delay, the minimum transmittance ratios and transmittance-affected region width were evaluated for all background–signal combinations, and their averages were used as representative values; the standard deviations of these values were taken as uncertainties. To further suppress residual large-scale intensity gradients in the normalized images, we fitted and subtracted a two-dimensional Gaussian profile before extracting the line profiles. For each corrected ratio image, we then considered the horizontal line through the center of the transmittance-affected area. We defined the lowest value of the line profile as the representative minimum value for each *Δt.* For spatiotemporal evolution, we calculated the x-ray transmittance-affected area width by setting 5% of the peak signal as the detection threshold and determining the width above this threshold for each time delay^31,32^.

### FLYCHK calculation

Charge-state distributions, x-ray transmittance, and radiation power were calculated using the collisional–radiative code FLYCHK developed by Chung et al.^44^ FLYCHK solves rate equations for atomic level populations in non-LTE plasmas. It employs a screened hydrogenic model and a super-configuration approach to treat many-electron ions efficiently. For a Ni plasma with an electron temperature of 120 eV, an average density of 13% of the solid state, and a thickness of 3.4 μm (taking into account the 28° incidence angle of the XFEL probe), the mean ionization state is *Z* ≈ 13.

It should be noted that the FLYCHK simulation did not reproduce the local wire-gap structure within the nanowire array at the early stage before hydrodynamic expansion of nanowire, because the initial condition was defined using the average density of the nanowire array. Therefore, local high density regions inside the nanowire array were not taken into account, which likely led to an underestimation of the inferred electron temperature at the earliest stage. Since the present experimental system cannot resolve the density structure of individual nanowires or its temporal evolution, this remains a limitation of the present study. Furthermore, the XFEL spectral bandwidth (≈ 30 eV) was not taken into account in the x-ray transmittance calculation.

### Measured and calculated electron temperature

We estimated *T*_e_ by comparing the minimum transmittance ratio in the measured line profile with the FLYCHK-calculated curve. In the FLYCHK calculations, we assumed a 3 μm thick Ni nanowire layer at 13% solid density (Fig. 3b). Because the curve has a minimum near *T*_e_ ≈ 160 eV, we could not determine *T*_e_ uniquely. We addressed this by constraining *T*_e_ ≤ 160 eV. If *T*_e_ >160 eV, the line profile would show both a decrease and an increase, whereas our measured value increased monotonically from the center to the edge. We therefore adopted *T*_e_ ≤ 160 eV and determined *T*_e_ accordingly.

### Nanowire array fabrication

We fabricated the nanowire array targets via an Anodic aluminum oxide (AAO) template-assisted method. AAO is a self-organized anodic alumina that has a honeycomb-like structure consisting of numerous nanopores. We used AAO as a template for nanowire growth. First, we anodized high purity aluminum (99.999%) to form AAO by applying a constant voltage in a phosphoric acid electrolyte, whose concentration was 0.1 M. Then, we sputtered a 100-nm-thin Au layer on the AAO bottom as a conductive layer. Subsequently, an electrochemical deposition process was conducted to form the nanowires and substrate. As explained in the Experimental setup section, the nanowires and substrate were made of different materials. We used a mixture of 1.1 M NiSO_4_•6H_2_O, 0.2 M NiCl_2_•6H_2_O, and 0.7M H_3_BO_3_ for the Ni nanowire growth, whereas we used a solution of 0.3 M CuSO_4_•5H_2_O and 0.01M H_2_SO_4_ for the Cu substrate formation. After the electrochemical deposition process, we dissolved the AAO template by soaking the samples with 2 M NaOH solution at 60 ℃. Then, we dried the nanowire array in a supercritical dryer to prevent nanowire agglomeration. More details regarding the fabrication process can be found in Ref 46.

### PIC simulations for the qualitative interpretation of the experimental results

**Fig. 8:**
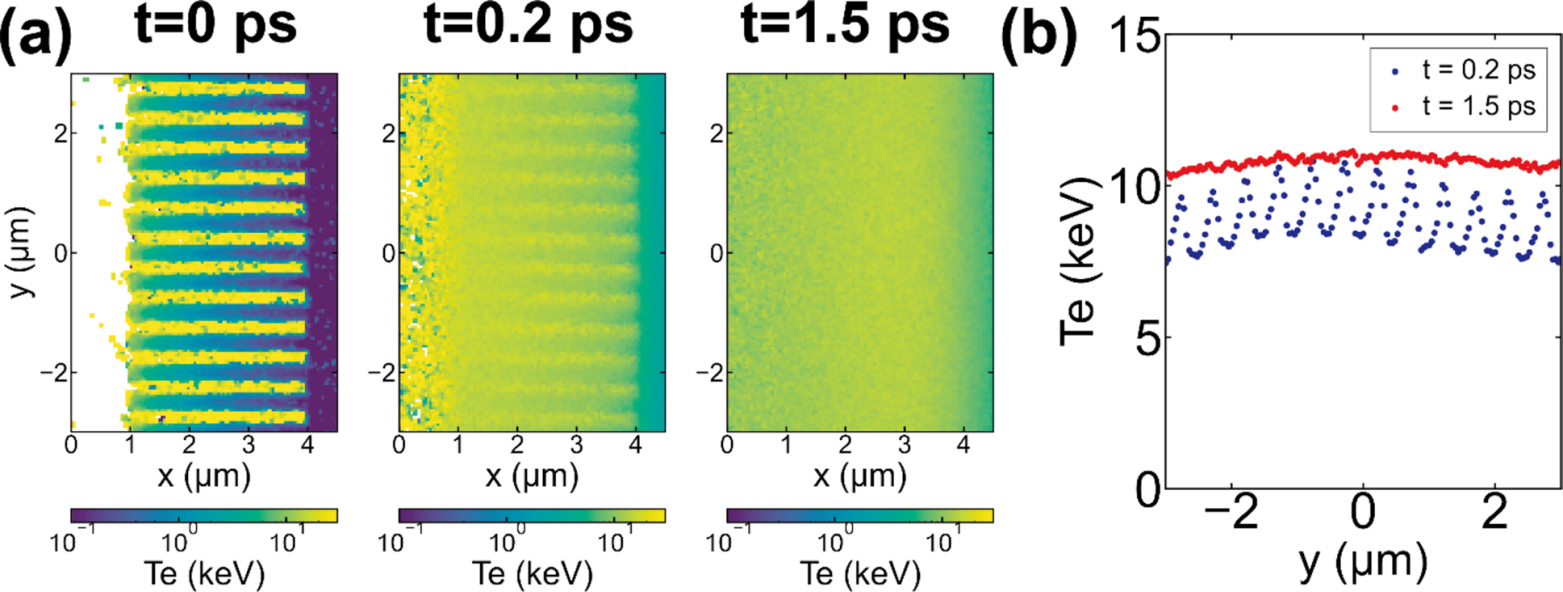
(**a**) Electron temperature distributions at *t* = 0, 0.2, and 1.5 ps calculated by PICLS-2D. (**b**) The averaged line profile for *t* = 0.2 (blue dot) and 1.5 ps (red dot). Each line profile was obtained from the cell-averaged electron temperature by taking the mean over the x-direction over the region where the wires are present (1 < *x* < 4).

We performed two-dimensional PIC simulations to qualitatively interpret the electron heating associated with nanowire array collapse. The calculations were performed using a two-dimensional particle-in-cell simulation code, PICLS-2D^48^. The laser had a spot size of 20 μm, a pulse duration of 30 fs, and an intensity of 3 × 10^18^ Wcm^− 2^. It was incident on the target at an angle of 45 °. The target was a 3-µm-long nanowire array on a 10-µm-thick substrate; the wires had a diameter of 200 nm and spacing of 500 nm. The simulation box covered an area of 60 × 50 μm, divided into 1500 × 1250 cells. The nanowire density was 64*n*_*c*_, and the mass ratio *M*_*i*_/*m*_*e*_ was 106,720, i.e., 58*M*_*p*_, where *n*_*c*_ is the critical density and *M*_*p*_ is the proton mass. The initial ionization state and temperature were set to two and zero, respectively. We did not include a pre-plasma in front of the target in the initial PIC simulation setup. The laser pulse was resolved in 180 timesteps. The time origin (*t* = 0 ps) was defined as the time at which the peak pulse reached the nanowire tips. Because of computational limitations, the simulations were performed for *t* = 1.6 ps. Figure 8a shows the simulated electron temperature distribution. At *t* = 0 ps, periodic features owing to the nanowire array were visible. As time progressed, these spatial modulations gradually disappeared, and a nearly uniform plasma state was reached. Figure 8b presents the line profiles of the electron temperature at *t* = 0.2 and 1.5 ps. These profiles were obtained from the cell-averaged electron temperature by taking the mean over the x-direction in the region 1 < *x* < 4, where the nanowire array was present. An increase in electron temperature was observed between *t* = 0.2 and 1.5 ps, indicating that the increase in electron temperature in the simulation was due to nanowire collapse. An increase in electron energy during nanowire collapse was also confirmed via an energy balance. However, the simulated electron temperature exceeded the experimentally evaluated temperature. If the plasma temperature reached a few keV, x-ray transmittance increased to 1.12 according to FLYCHK calculation. However, we did not observe the x-ray transmittance increase in Ni nanowires in the center of the transmittance-affected area, and this means that PIC simulations overestimated the electron temperature of nanowires. The pre-plasma scale length was estimated using the one-dimensional radiation–hydrodynamics code ILESTA-1D^49^. This calculation was performed in planar geometry using the measured pre-pulse temporal profile. The result shows that a pre-plasma layer of approximately 1 μm is formed in front of the target surface, 1 ps before the arrival of the main pulse. The corresponding critical-density surface is located ~ 0.34 μm from the initial solid surface. The 1D simulation also indicated that the region along the laser-propagation direction where the surface is significantly compressed and ablated extends only to a depth of ~ 170 nm from the initial surface, which is much smaller than the nanowire length (~ 3 μm). This suggests that the tips of the nanowires are deformed by the pre-pulse. The presence of this pre-plasma reduces the effective energy coupling to the nanowire array in the experiment and is therefore a possible factor underlying the discrepancy between the simulated and measured results.

## Data Availability

The data that support the findings of this study are available from the corresponding author upon reasonable request.
